# Resolution enhancement of electrical resistance tomography by iterative back projection method

**DOI:** 10.1007/s12650-015-0308-8

**Published:** 2015-08-12

**Authors:** Noriaki Ichijo, Shinsuke Matsuno, Taiji Sakai, Yoshikatsu Tochigi, Meguru Kaminoyama, Kazuhiko Nishi, Ryuta Misumi, So Nishiyama

**Affiliations:** IHI Corporation, 1 Shin-nakahara-cho, Isogo-ku, Yokohama, 235-8501 Japan; Division of Materials Science and Chemical Engineering, Faculty of Engineering, Yokohama National University, 79-5 Tokiwadai, Hodogaya-ku, Yokohama, 240-8501 Japan

**Keywords:** Electrical resistance tomography, Image reconstruction, Back projection, Glass melter, Iterative algorithm

## Abstract

**Abstract:**

An iterative back projection method (i-BP) has been developed to improve the resolution of reconstructed images produced by electrical resistance tomography (ERT). This solution is based on an iterative calculation of the electrical fields and it is possible to reconstruct clearer images than those reconstructed by the conventional back projection method without divergence. However, it does take several minutes to finish the iteration process, and therefore this solution can be applied to flow fields that require high spatial resolution rather than short processing times, such as the accumulation of noble metals in glass melters. Numerical simulations and experiments using a simple model are performed in this study. The numerical simulations show that clear images are reconstructed both near the wall and at the center by i-BP. The conductivity correlation coefficient between the genuine distribution and the reconstructed image is improved from 0.4 to 0.9. The validity of the i-BP method is also confirmed by the experimental results. As a result, it is confirmed that ERT and i-BP are capable of reconstructing acceptable images and have potential for use in the visualization of the accumulation of noble metals in a glass melter.

**Graphical Abstract:**

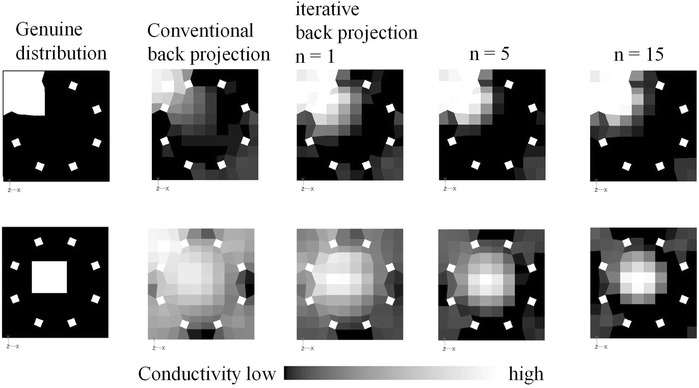

## Introduction

**Fig. 1:**
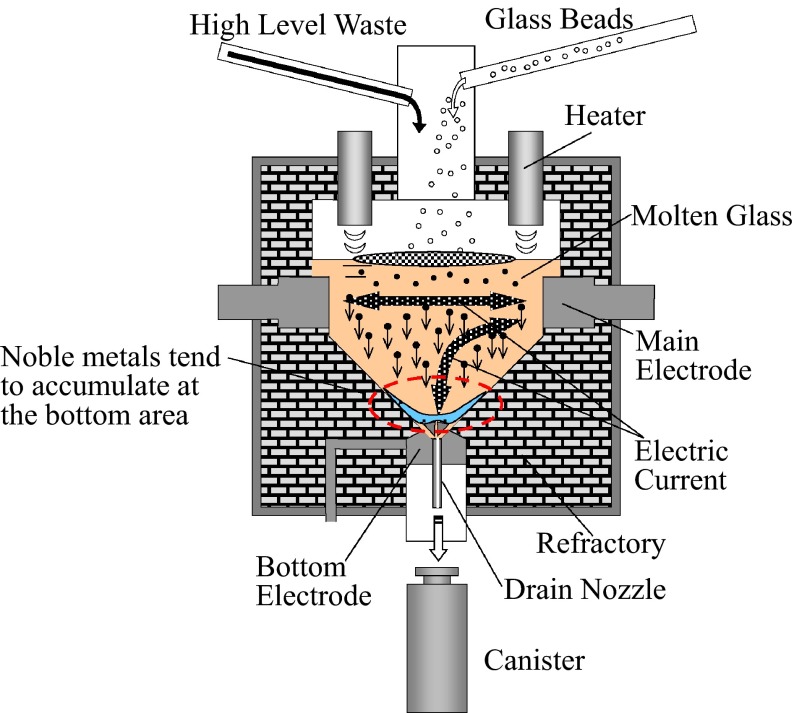
High-level radioactive liquid waste glass melter

Joule-heated glass melters are used for vitrification processing of the high-level radioactive liquid waste (HLW) generated during the nuclear fuel cycle. The outline of a glass melter is shown in Fig. 1. The width and the height of the vessel are both approximately 1.5 m. In this process, the HLW and the glass material (glass beads) are melted by Joule heating and mixed inside the melter. The molten glass is then drained into a metal canister for the next stage of the storage process.

Throughout the process, the behavior of the noble metals is very important for stable operation of the melter, because Joule heating is strongly influenced by the high electrical conductivity of these noble metals. To clarify the behavior and accumulation of the noble metals, many simulation studies have been performed (Matsuno et al. 2008; Iso et al. 2008). The numerical results agreed well with the experimental data in terms of the temperature distributions and electrical resistance values. However, in-situ observation of the behavior of the noble metals in the glass melter is strongly required to ensure an efficient melting process.

Electrical resistance tomography (ERT) has previously been applied to many industrial processes, including a suspension polymerization reactor (Kaminoyama et al. 2005, 2010), a gas–liquid two-phase flow in a pipe (Ma et al. 2001), and a stirred tank (Mann et al. 1997). Other electrical tomographic techniques use capacitance (Corlett 1999) and electrostatic charge (Machida and Kaminoyama 2008). These conventional methods are exclusively applied to cylindrical sections and electrodes that are installed on a wall. In contrast, the glass melter has a non-circular section and it is possible to insert the electrodes from the top of the melter to inspect the local accumulation. Furthermore, the information of the inside the melter is temperatures and resistivity of some limited points. Therefore, requirements of ERT are not only the resolution but also the reliability of the reconstructed image with fewer errors. When considering these measurement conditions, the following problems must be solved:

1. application to a rectangular section
2. clarification of a suitable image reconstruction algorithm.

A previous study (Ichijo et al. 2012) has shown that conventional methods (data acquisition methods; the adjacent method, image reconstruction algorithms; the back projection method) can be applied to a rectangular section and better images were obtained when the electrode arrangement was circular. However, the clarity of the images was not sufficient to detect the accumulation of the noble metals, especially at the center of the vessel. Therefore, developing a high resolutional image reconstruction algorithm is required.

In this study, a suitable image reconstruction algorithm for application to a glass melter is proposed. Many image reconstruction algorithms have been proposed previously, including the back projection method (Kotre 1989), the Tiknohov regularization method (Vauhkonen et al. 1998), and the modified Newton–Raphson method (MNR method) (Yorkey et al. 1987), among others. When focusing on the application to glass melters, the spatial resolution takes priority over a high sampling rate because of the low flow velocity (less than 10^−3^ m/s) inside the glass melter. In addition, erroneous images must not be reconstructed to prevent misoperation of the glass melter. When these requirements are considered, the conventional back projection method (CBP) does not have sufficient resolution and the MNR method has a risk of divergence in its iterative calculations. Therefore, this study describes the development of a new iterative image reconstruction algorithm, the iterative back projection method (i-BP), with particular focus on high-resolution imaging and the stability of the iterative calculations.

## ERT theory and method

### Image reconstruction algorithm

The ERT technique is used to visualize the distribution of a multiphase flow based on the electrical conductivity difference between two substances. In a glass melter, ERT visualizes the distribution of the noble metals that are contained in the molten glass.

**Fig. 2:**
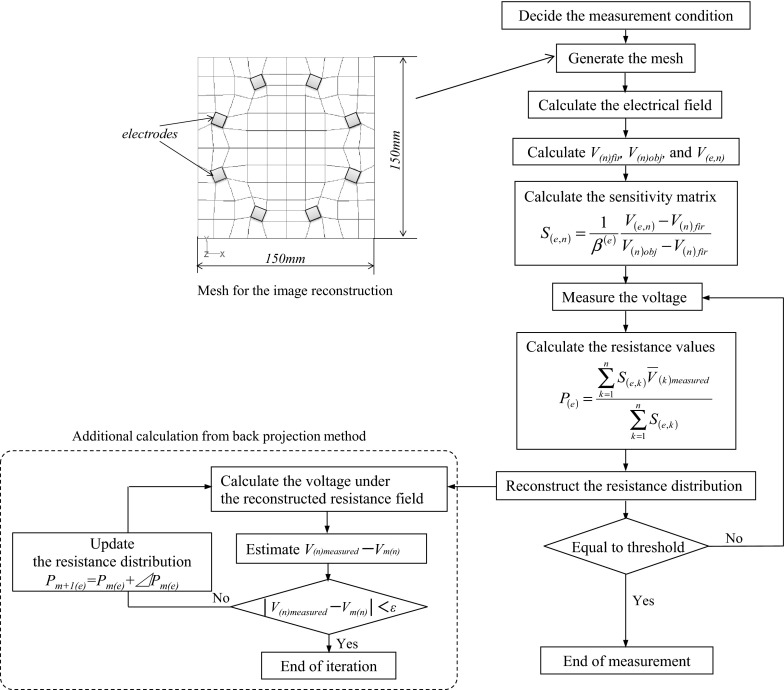
Measurement flows of ERT and i-BP

CBP and i-BP are used as the reconstruction algorithms. The measurement flow is shown in Fig. 2. I-BP is Algebraic Reconstruction Technique same as CBP. The measurement flow of the back projection method is shown on the right side of Fig. 2, while the left side shows the additional calculation flow for i-BP. *V*_(*n*)fir_ is the measured voltage when the entire area is filled with a lower resistance substance, *V*_(*n*)obj_ is the measured voltage when the entire area is filled with a higher resistance substance, *V*_(*e, n*)_ is the measured voltage when the *e*th element is filled with the higher resistance substance and the other elements are filled with the lower resistance substance, *β*^(*e*)^ is the fractional area of the *e*th element, *P*_(*e*)_ is the equivalent resistance value, and *V*_(*k*)_ is the measured voltage obtained experimentally. Before the voltage is measured, the sensitivity matrix *S*_(*e, n*)_ is calculated by the finite element method (FEM). After the calculation is performed, the resistance distribution is reconstructed based on the sensitivity matrix and the measured voltage. $\overline{V}_{{ (k){\text{measured}}}}$ is the normalized voltage calculated using Eq. (1):

1 $$ \overline{V}_{{\left( k \right){\text{measured}}}} = \frac{{V_{{\left( k \right){\text{measured}}}} - V_{{\left( k \right){\text{fir}}}} }}{{V_{{\left( k \right){\text{obj}}}} - V_{{\left( k \right){\text{fir}}}} }} $$

The measurement is terminated by using a measurement time or a measurement count. For the iterative algorithm, an electrical calculation is implemented for the resistance field that was estimated by the back projection method. The boundary condition for one of the current injecting electrodes is +*I*, while the other is −*I*; the voltage is then calculated under the same conditions as those used in the experiment and 20 voltage measurement values are obtained. If the difference between the calculated and experimental values is below the threshold value “ε”, then the iteration is complete. If not, the iteration continues and updates the resistance distribution. The updated value is calculated based on the sensitivity coefficient and the difference between the voltage measurement value from the experiments and that from the calculations. The updated resistance value *P*_*m*+*1*(*e*)_ is defined as

2 $$ P_{m + 1(e)} = P_{m(e)} + \Delta P_{m(e)} $$

where $\Delta P_{m(e)}$ is given by

3 $$ \Delta P_{m\left( e \right)} = \sum\limits_{k = 1}^{n} {\left( {V_{{(k){\text{measured}}}} - V_{m(k)} } \right) \cdot S_{(e,k)} } $$

where $V_{{(k){\text{measured}}}}$ is the *k*th voltage value measured experimentally and $V_{m(k)}$ is the *k*th voltage value estimated by the *m*th iteration. The iteration process only uses electric field calculations based on Poisson’s equation, and does not use an ill-conditioned matrix like Hessian matrix. In addition, the resistivity distribution obtained by back projection is used as an initial condition of the iterative calculation, therefore, the number of iteration can be reduced and i-BP can obtain a stable solution.

### Data acquisition method

**Fig. 3:**
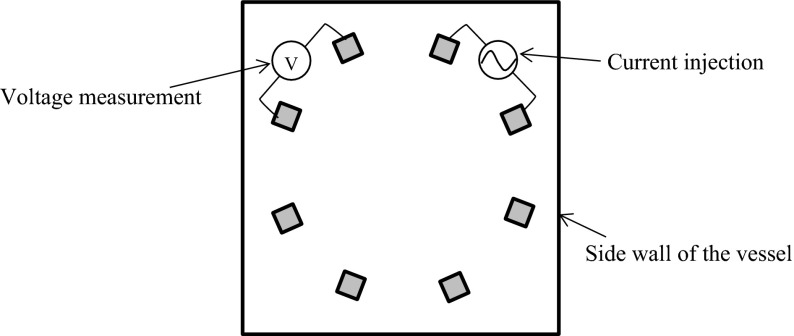
Adjacent data acquisition method

The data acquisition method used here is the adjacent method (Dickin and Wang 1996; Williams and Beck 1995), which is shown in Fig. 3. A current is injected through two neighboring electrodes and the voltages are measured by other pairs of neighboring electrodes. After the voltages are measured, a current is then injected through the next pair of electrodes and the voltage measurement process is repeated until all independent measurements have been completed. In the adjacent method, the total number of independent measurements is calculated using Eq. (4).

4 $$ M = N\left( {N - 3} \right)/2 $$

where *M* is the total number of independent measurement values and *N* is the number of electrodes. In the case where *N* = 8, *M* is calculated to be 20.

## Numerical simulation

### Calculation method

The numerical simulation model has the same section size as the experimental vessel that was described in Sect. 4.1. A steady-state 2D calculation is implemented to reduce the calculation time. A 2D calculation can ignore the effects of the bottom wall and the liquid surface, and thus this simulation focuses on evaluation of the image reconstruction algorithm. The electrode arrangement is circular, as shown in Fig. 3. The electrical field is calculated using the general-purpose simulation code ANSYS Fluent ver. 12.1.4. The electrical field is solved using a user-defined function, and the basic equation for the electrical potential is defined as

5 $$ \nabla \left( {\sigma \cdot \nabla \varphi } \right) = 0 $$

where *σ* (S/m) is the electrical conductivity and *φ* (V) is the electrical potential. The current is injected between neighboring electrodes as the boundary condition, and the electrical potentials between the other neighboring electrodes are calculated. The numerical conditions are summarized in Table 1. The electrical conductivity and the temperature are almost in the same range as those quantities in the actual glass melter.

**Table 1:** Numerical conditions

Calculational parameter	
Temperature (°C)	1000
Electrical conductivity (molten glass) (S/m)	8.6
Electrical conductivity (molten glass containing noble metals) (S/m)	36.4
Conductivity ratio (with/without noble metals) (–)	4.2
Electric current (mA)	85

### Results and discussion

**Fig. 4:**
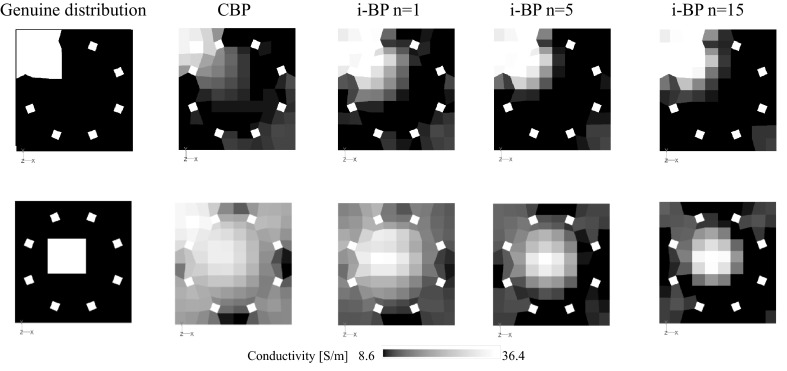
Reconstructed images (effect of iterative method)

**Fig. 5:**
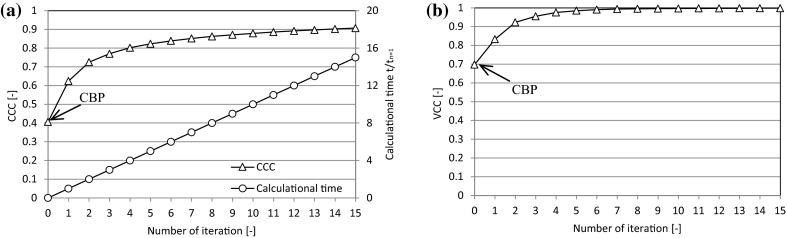
Improvement in a CCC and b VCC by using i-BP when the high conductivity substance is at the corner t_n=1_ on the vertical axis is the calculation time for the 1st iteration

Conductivity images showing genuine distributions and images that were reconstructed by CBP and i-BP are shown in Fig. 4. When compared with the genuine distributions, the images that were reconstructed by CBP are blurred and the resolution is low, especially when the high conductivity substance is located at the center of the vessel. In contrast, images that were reconstructed by i-BP become clearer at both the corner and the central region, and the more that the iteration is repeated, then the clearer the reconstructed image becomes. To confirm the effect of i-BP quantitatively, the reconstructed images and the voltage measurement values are evaluated using Pearson’s correlation coefficient (Pearson, 1895). The conductivity correlation coefficient (CCC) and the voltage correlation coefficient (VCC) are given by

6 $$ {\text{CCC}} = \frac{{\sum\nolimits_{e = 1}^{n} {\left( {C_{r\left( e \right)} - \overline{{C_{r} }} } \right)\left( {C_{i\left( e \right)} - \overline{{C_{i} }} } \right)} }}{{\sqrt {\sum\nolimits_{e = 1}^{n} {\left( {C_{r\left( e \right)} - \overline{{C_{r} }} } \right)^{2} } } \sqrt {\sum\nolimits_{e = 1}^{n} {\left( {C_{i\left( e \right)} - \overline{{C_{i} }} } \right)^{2} } } }} $$

7 $$ {\text{VCC}} = \frac{{\sum\nolimits_{k = 1}^{n} {\left( {V_{r\left( k \right)} - \overline{{V_{r} }} } \right)\left( {V_{i\left( k \right)} - \overline{{V_{i} }} } \right)} }}{{\sqrt {\sum\nolimits_{k = 1}^{n} {\left( {V_{r\left( k \right)} - \overline{{V_{r} }} } \right)^{2} } } \sqrt {\sum\nolimits_{k = 1}^{n} {\left( {V_{i\left( k \right)} - \overline{{V_{i} }} } \right)^{2} } } }} $$

where *C*_*r*(*e*)_ is the electrical conductivity of the *e*th element of the genuine distribution, *C*_*i*(*e*)_ is the electrical conductivity of the *e*th element at the *i*th iteration of the reconstructed image, *V*_*r*(*k*)_ is the *k*th experimental voltage value, *V*_*i*(*k*)_ is the *k*th voltage value at the ith iteration, and $\overline{{C_{r} }}$, $\overline{{C_{i} }}$, $\overline{{V_{r} }}$ and $\overline{{V_{i} }}$ are arithmetic mean values. The correlation coefficient can be interpreted thus: 1 is perfect, 0.7–0.9 is strong, 0.4–0.6 is moderate, 0.1–0.3 is weak and 0 represents no correlation (Dancey and Reidy 2004). The CCC is improved significantly by i-BP, as shown in Fig. 5, to 0.9 after 15 iterations, while the CCC is rather low at 0.4 when using CBP, which is equivalent to the case where iteration = 0. Similarly, the VCC increases with repeated iterations. The correlation coefficient is effectively improved, especially at the early stage of the iterations, but the calculation time is proportional to the number of iterations, as shown in Fig. 5a. The calculation time depends on the performance of the computer, and it takes within 2 min to complete one iteration by a typical desktop computer with 2 GB of RAM. Although 20–30 min are required to complete 15 iterations, clear images can be obtained after even a few iterations. Therefore, the actual calculation time to obtain the acceptable reconstructed image is normally within 10 min.

Other image reconstruction algorithms requires less calculation time, for example, CBP requires within one second and MNR requires within 1 min (Yorkey et al. 1987). However, when the flow in the glass melter is considered, the calculation time of i-BP is acceptable.

## Experimental

### Experimental method

**Fig. 6:**
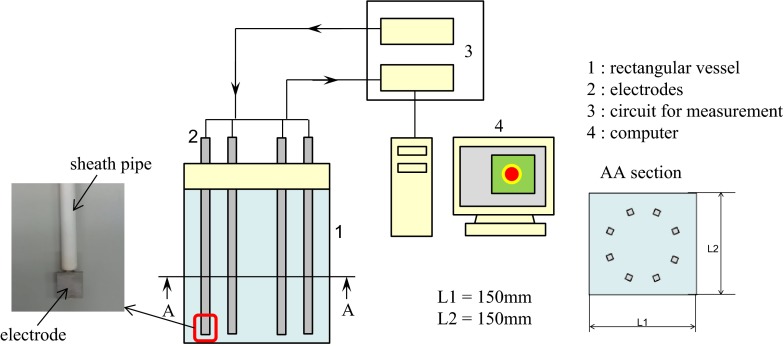
Experimental apparatus

The experimental apparatus is shown in Fig. 6. The vessel is 150 mm wide, 150 mm long and 200 mm high. The electrodes are 3 mm in diameter and these electrodes are covered with a sheath pipe made from aluminum oxide, except at the lower sections. This sheath pipe insulates the upper parts of the electrodes. At the lower section of the electrode, a 10 mm × 10 mm stainless steel piece is welded to increase the surface area. The electrode arrangement is circular, which is the same as the arrangement in the numerical simulation. The image reconstruction methods used are CBP and i-BP.

**Fig. 7:**
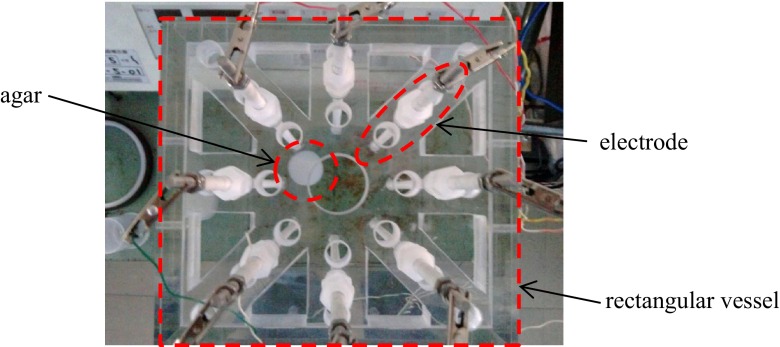
Experimental setup (*top view*)

The experimental conditions are summarized in Table 2. In this experiment, a sodium chloride solution made from a higher concentration sodium chloride solution and agar are used as the background fluid and as a simulant of the noble metal accumulation, respectively. However, the electrical conductivity of molten glass contained the noble metals of 1000° is much higher than that of a saturated sodium chloride solution of normal temperature, therefore, only the electrical conductivity ratio is adjusted based on the calculational conditions, as shown in Table 1, and the actual electrical conductivity value of background fluid is 3.0 S/m, which is three times lower than that of molten glass. Instead, the injecting electrical current is 20 mA, therefore, voltage levels of the numerical simulation and the experiments are almost the same. Figure 7 shows an example of the measurement setup.

**Table 2:** Experimental conditions

Experimental parameter	
Electrical conductivity (sodium chloride solution) (S/m)	3.0
Electrical conductivity (agar made of sodium chloride solution) (S/m)	12.6
Conductivity ratio (with/without noble metals) (–)	4.2
Electric current (mA)	20

### Results and discussion

**Fig. 8:**
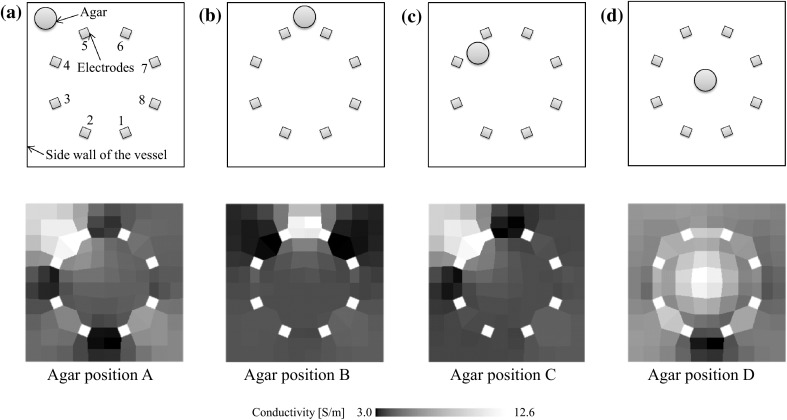
Reconstructed images of experiments using CBP, a agar position A, b agar position B, c agar position C, d agar position D

**Fig. 9:**
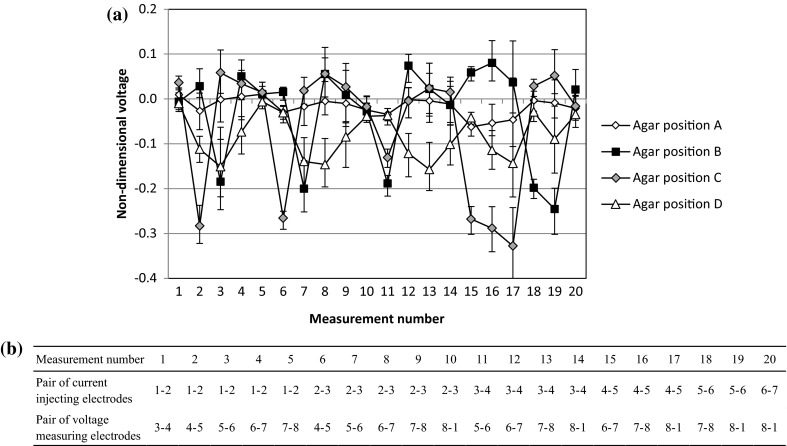
a Voltage measurement values at each agar position; b current injection electrodes and voltage measurement electrodes for each measurement number

The agar positions and the images reconstructed by CBP are shown in Fig. 8. Agar, which has high conductivity, can be detected in the case where it is located near the electrodes (agar positions B and C). In contrast, when agar is at the corner of the vessel (agar position A), the reconstructed image misrecognizes the presence of agar between two electrodes. Also, when agar is at the center (agar position D), the reconstructed image recognizes the correct position, but the contrast becomes too low. This shows similar tendencies to the numerical simulation results. To analyze the reasons for such low resolution, the non-dimensional voltage V_(n)_ and the average of absolute values of non-dimensional voltages $\overline{V}$ are defined as

8 $$ V_{(n)} = \frac{{v_{{(n){\text{agar}}}} - v_{{(n){\text{NaCl}}}} }}{{v_{{(n){\text{NaCl}}}} }} $$

9 $$ \overline{V} = \sum\limits_{n = 1}^{20} {\left| {V_{(n)} } \right|} $$

where v_(n)agar_ is the experimental voltage when agar is in the sodium chloride solution and v_(n)NaCl_ is the experimental voltage when no agar is in the sodium chloride solution. A larger absolute value for the non-dimensional voltage indicates high sensitivity towards the agar position. The numbers from 1 to 20 along the horizontal axis match the measurement numbers in Fig. 9b. Table 3 shows the average and maximum values of the absolute non-dimensional voltages in each agar position. The absolute values of the non-dimensional voltages of agar positions B and C are much larger than those of agar positions A and D. The average of the absolute values of the non-dimensional voltages in agar position C is five times larger than that in agar position A as shown in Table 3. This means that the distance between the agar and the electrodes has an effect on the sensitivity of the voltage measurement values. In agar position D, the absolute maximum value is smaller than that of agar position B and C, however every measurement pattern has a little sensitivity. Therefore, the average is larger than that in agar position A. As a result, the reconstructed image of agar position D is blurred.

**Table 3:** The average and maximum values of the absolute non-dimensional voltages in each agar position

Agar position	A	B	C	D
Maximum $\left| {V_{(in)} } \right|$	0.06	0.25	0.33	0.16
$\overline{{V_{(in)} }}$	0.02	0.08	0.10	0.08

**Fig. 10:**
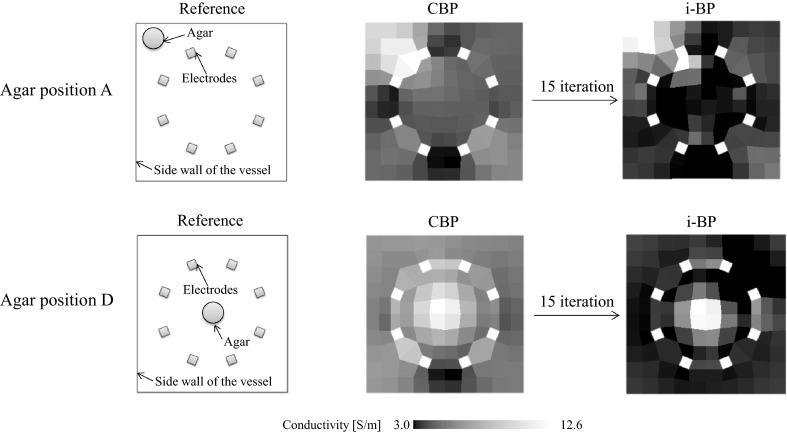
Reconstructed images of experiments produced by i-BP

**Fig. 11:**
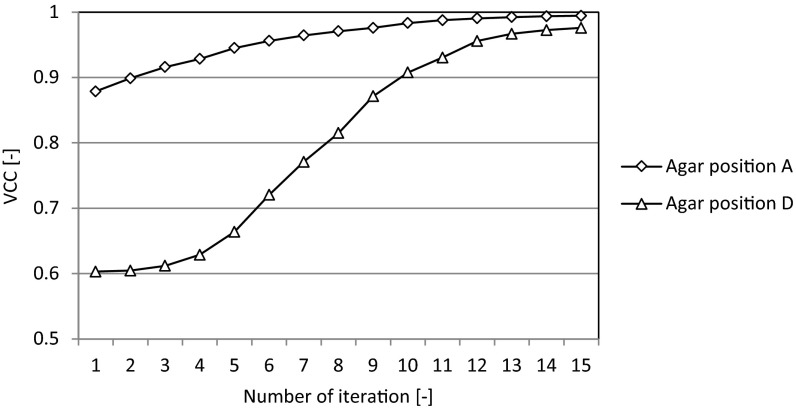
VCC from i-BP in agar position A and D

To improve the resolution for agar positions A and D, i-BP is applied. The agar positions and the images that were reconstructed by the CBP and i-BP are shown in Fig. 10. The reconstructed images produced by i-BP are much clearer than those produced by the CBP at both agar positions A and D. When the agar is at the center, the contrast of the reconstructed image is effectively improved. VCC becomes closer to 1.0 as the iterative calculation is repeated, as shown in Fig. 11. However, there is an error element near the electrode in the case of agar position A. This error may be caused not only by measurement errors but also by the sensitivity matrix. In this experiment, the sensitivity matrix is estimated by a 2D model and not by a 3D model to shorten time required for the whole process. Therefore, the effects of the bottom wall of the vessel and the liquid surface cannot be considered. The sensitivity matrix calculated by 3D model has a potential to minimize both the error and the noise. In addition, increasing the value of the electrical current is also effective in enlarging both the voltage value and the signal-to-noise ratio.

## Conclusion

In this study, numerical simulations and experiments are performed to demonstrate a new reconstruction algorithm called i-BP. The numerical simulations focus on development of the image reconstruction algorithm, and i-BP can obtain better reconstructed images than the CBP. The experiments also reveal the validity of i-BP. Consideration of these results shows that ERT and i-BP have the potential to be used to visualize the distribution of the noble metals and molten glass inside of a glass melter.

## References

[CR1] Corlett AE (1999). Three-phase flow pattern recognition in horizontal pipelines using electrical capacitance tomography. J Vis.

[CR2] Dancey C, Reidy J (2004). Statistics without maths for psychology: using SPSS for windows.

[CR3] Dickin FJ, Wang M (1996). Electrical resistance tomography for process applications. Meas Sci Technol.

[CR4] Ichijo N, Matsuno S, Tokura S, Tochigi Y, Kaminoyama M, Nishi K, Misumi R, Higashi K (2012) Applicability of electrical resistance tomography to monitoring noble metal accumulation in glass melter. In: 6th International symposium on process tomography (ISPT6), Cape Town PO16

[CR5] Iso Y, Matsuno S, Uchida H, Oono I, Fukui T, Ooba T (2008). Numerical simulation of platinum group metal particles behaviour in a Joule-heated glass melter. J Power Energy Syst.

[CR6] Kaminoyama M, Taguchi S, Misumi R, Nishi K (2005). Monitoring stability of reaction and dispersion states in a suspension polymerization reactor using electrical resistance tomography measurement. Chem Eng Sci.

[CR7] Kaminoyama M, Kato K, Misumi R, Nishi K (2010). Measurements of the phase inversion phenomenon in a suspension polymerization reactor with an electrical resistance tomography system. J Chem Eng Jpn.

[CR8] Kotre CJ (1989). A sensitivity coefficient method for the reconstruction of electrical impedance tomograms. Clin Phys Physiol Meas.

[CR9] Ma Y, Zheng Z, Xu L, Liu X, Wu Y (2001). Application of electrical resistance tomography system to monitor gas/liquid two-phase flow in a horizontal pipe. Flow Meas Instrum.

[CR10] Machida M, Kaminoyama M (2008). Sensor design for development of tribo-electric tomography system with increased number of sensors. J Vis.

[CR11] Mann R, Dickin FJ, Wang M, Dyakowski T, Williams RA, Edwards RB, Forrest AE, Holden PJ (1997). Application of electrical resistance tomography to interrogate mixing process at plant scale. Chem Eng Sci.

[CR12] Matsuno S, Iso Y, Uchida H, Oono I, Fukui T, Ooba T (2008). CFD Modeling Coupled with Electric Field Analysis for Joule-Heated Glass Melters. J Power Energy Syst.

[CR13] Pearson K (1895). Notes on regression and inheritance in the case of two parents. Proc R Soc Lond.

[CR14] Vauhkonen M, Vadasz D, Kaipio JP, Somersalo E, Karjalainen PA (1998). Tikhonov regularization and prior information in electrical impedance tomography. IEEE Trans Med Imaging.

[CR15] Williams RA, Beck MS (1995) Process tomography: principles techniques and applications. Butterworth-Heinemann, Oxford. ISBN 0750607440

[CR16] Yorkey TJ, Webster JG, Tompkins WJ (1987). Comparing Reconstruction Algorithms for Electrical Impedance Tomography. IEEE Trans Biomed Eng.

